# Effect and Mechanism of ShiZhiFang on Uric Acid Metabolism in Hyperuricemic Rats

**DOI:** 10.1155/2018/6821387

**Published:** 2018-06-25

**Authors:** Yansheng Wu, Yixing Wang, Jiaoying Ou, Qiang Wan, Liqiang Shi, Yingqiao Li, Fei He, Huiling Wang, Liqun He, Jiandong Gao

**Affiliations:** ^1^Department of Nephrology, Shuguang Hospital Affiliated to Shanghai University of Traditional Chinese Medicine; TCM Institute of Kidney Disease of Shanghai University of Traditional Chinese Medicine; Key Laboratory of Liver and Kidney Diseases (Shanghai University of Traditional Chinese Medicine), Ministry of Education; Shanghai Key Laboratory of Traditional Chinese Clinical Medicine (14DZ2273200), No. 528 Road Zhangheng, Shanghai 201203, China; ^2^Department of Internal Medicine of Traditional Chinese Medicine, Shanghai East Hospital, Tongji University School of Medicine, No. 150 Road Jimo, Pudong New District, Shanghai 200120, China; ^3^Department of Internal Medicine, Shanghai TCM-Integrated Hospital, Affiliated to Shanghai University of Traditional Chinese Medicine, No. 184 Road Baoding, Shanghai 200082, China; ^4^Department of Nephrology, Traditional Chinese Medicine Hospital of Langfang City, No. 108 Road North Yinhe, Langfang 065000, China; ^5^Department of Nephrology, Xiamen Hospital of Traditional Chinese Medicine, No. 1739 Road Xianyue, Xiamen 361009, China

## Abstract

**Objective:**

To explore the effect and mechanism of ShiZhiFang on uric acid metabolism.

**Methods:**

40 rats were divided into normal group, model group, ShiZhiFang group, and benzbromarone group. The hyperuricemic rat model was induced by yeast gavage at 15 g/kg and potassium oxonate intraperitoneal injection at 600 mg/kg for two weeks. During the next two weeks, ShiZhiFang group rats were given ShiZhiFang by gavage, and benzbromarone group rats were given benzbromarone by gavage. The serum uric acid, creatinine, blood urea nitrogen, XOD activity, urinary uric acid, urinary *β*_2_-MG, and histopathological changes were observed in the rats of each group after treatment.

**Results:**

The hyperuricemic model was established successfully and did not show the increase of serum creatinine and blood urea nitrogen. Compared with the model group, the serum uric acid, serum XOD activity, and urinary *β*_2_-MG were significantly decreased (*p* < 0.05), and 24 h urinary uric acid excretion was significantly decreased (*p* < 0.01) in ShiZhiFang group, whereas the two treatment groups were of no statistical significant in above indicators (*p* > 0.05); renal histopathology showed that the lesions in two treatment groups were reduced compared to the model groups. The gene and protein expression of uric acid anion transporters rOAT1 and rOAT3 in the kidney was significantly higher than that in model group (*p* < 0.01).

**Conclusion:**

The model is suitable for the study of primary hyperuricemia. The mechanisms of ShiZhiFang on uric acid metabolism in hyperuricemic rats may be involved in reducing the activity of serum XOD and promoting the transcription and expression of rOAT1 and rOAT3 in the kidney.

## 1. Introduction

Hyperuricemia (primary hyperuricemia) is caused by abnormal purine metabolism or decreased uric acid excretion. 2/3 uric acid in the human body comes from the liver, muscles, and intestines, and 1/3 uric acid comes from diet containing rich purine, such as seafood and meat [1]. Xanthine oxidase (XOD) is a key enzyme in the synthesis of uric acid. Xanthine is formed by xanthine oxidase catalyzed hypoxanthine and generated by guanine deaminase catalyzed guanine, while the xanthine oxidase oxidizes xanthine again to form the final product: uric acid [2]. Therefore, the activity of XOD determines the production of uric acid. The kidney is an important organ that maintains uric acid balance in the body. 90% uric acid is reabsorbed into the blood through the renal tubule and 2/3 uric acid is excreted through the kidneys [3]. Renal excretion disorder is the main cause of hyperuricemia. The transporter located in the proximal tubule epithelial cells makes the intracellular anion exchange with uric acid and plays the role of secreting and reabsorption of uric acid. The reabsorption of uric acid is almost done at the S1 end of the proximal tubule, but the proximal end of the tubular S2 has double roles of reabsorption and secretion of uric acid, but it is mainly responsible for the secretion of uric acid [4]. Accordingly, the uric acid transporter in the renal proximal tubule plays an important role in regulating the level of serum uric acid, and the function impairment of the uric acid transporter in epithelial cells can cause uric acid transport disorder.

Human organic anion transporters (OAT), OAT1 and OAT3, belong to the SLC22A family and contributed to transportation of uric acid in the kidney, especially in the secretion and excretion of uric acid. In the kidney, a variety of endogenous and exogenous toxins and drugs are excreted in the urine as well as through the organic anion transporters. When the anion transporters are impaired, urate excretion decreases, renal tubular cells are impaired, concentration is dysfunctional, and microprotein is leaking from the kidneys. Beta-2-microglobulin (*β*2-MG) is a low molecular protein that can be filtered by the glomerulus and almost completely reabsorbed by the renal tubules. In the case of mild renal tubular injury, *β*2-MG production and glomerular filtration rate is normal, while excessive *β*2-MG is excreted from the urine, pointing out that tubule concentration is dysfunctional and renal tubular cells have been damaged [5].

Hyperuricemia is closely related to the occurrence and development of various diseases. According to a meta-analysis and systematic review [6] of uric acid levels and mortality in patients with chronic kidney disease (CKD), high levels of uric acid are closely related to mortality, and the death risk increases by 8% when every 1mg/ml uric acid was increased. Uric acid is also a risk factor of cardiovascular disease. Uric acid levels are closely related to hypertension, coronary heart disease events, heart failure, and atrial fibrillation and especially are positively related to the deterioration and death of systolic heart failure [7–9]. In addition, uric acid is also closely related to insulin resistance [10] and hyperlipidemia. Therefore, it is necessary to control uric acid level. The European guidelines suggest that serum uric acid level in patients with chronic hyperuricemia should be maintained at ≤6mg/dl, showing that diet is the key to the treatment of hyperuricemia but it is indispensable to rationally use drugs to achieve the ideal control effect of uric acid [11]. At present, the main treatment of hyperuricemia is allopurinol that is a classical xanthine oxidase inhibitor, benzbromarone that promotes the excretion of uric acid, and febuxostat that is a novel xanthine oxidase inhibitor; however, these drugs can temporarily alleviate the symptoms, but more adverse reactions follow [12]. Traditional Chinese Medicine (TCM) treatment for hyperuricemia has gradually attracted attention, and its efficacy has shown many advantages. Many studies have shown that the TCM formula, single drug, and active ingredients in single herb have obvious effect of reducing uric acid [13–17]. TCM formula of ShiZhiFang (SZF) is composed of plantago seeds, white mustard seeds, vaccaria seeds, and abutilon seeds, which has been used more than 20 years in Shuguang Hospital (Shanghai, China), and confirmed the clinical safety and efficacy of decreasing uric acid [18]. The purpose of this study is to investigate the regulatory effect and related mechanism of SZF on uric acid metabolism in hyperuricemic rats.

## 2. Materials and Methods

### 2.1. Experimental Animals

Forty male Sprague–Dawley (SD) rats of SPF grade, of weight 190±10g, were purchased from Shanghai Slaccas Experimental Animal Co. Ltd. Rats were raised in the experimental animal center of Shanghai University of Traditional Chinese Medicine. All animal experiments were approved by the ethics committee of the laboratory animal center of Shanghai University of Traditional Chinese Medicine, Safety Certificate Number: SCXK (Shanghai) 2008-0016.

### 2.2. Drugs and Reagents

Benzbromarone (0.05 g, Lot No. 2901, Xinyi Wanxiang Pharmaceutical Limited by Shares Ltd., Shanghai, China) was prepared with physiological saline into suspension, and the concentration was 55.33mg/ml. Yeast powder (Lot No. OXOID-L21, OXOID, United Kingdom) was made from distilled water into 2g/ml pulp. Potassium oxonate (Lot No. 080601, Zhongke Taidou Chemical Reagent Co. Ltd., Shandong, China) was dissolved in 0.9% sodium chloride injection into 300mg/ml concentration. XOD test kits (No. A002) were purchased from Nanjing Jiancheng Biological Engineering Institute (Nanjing, China). Rabbit anti-rat OAT1 (Lot No. YT3320) antibody was purchased from Immunoway (United States). Rabbit anti-rat OAT1 (SLC22A6 YT3320) antibody was purchased from Immunoway (United States). Mouse anti-rat OAT3 (SLC22A8 H00009376-B01P) antibody was purchased from NOVUS (United States). PrimeScriptTM RT reagent kit with gDNA Eraser (No. RR047A) and SYBR Premix Ex TaqTM (No. RR420A) were all from TaKaRa (Japan).

### 2.3. SZF Fingerprint Identification

**Figure 1:**
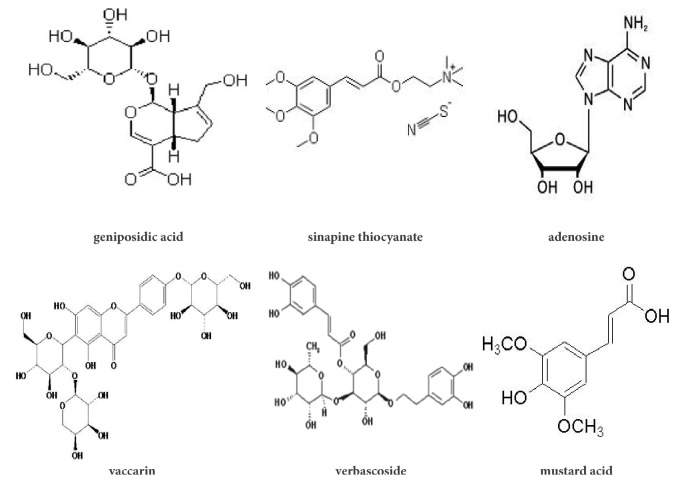
Main composition of SZF.

SZF Fingerprint Identification is accomplished by Shanghai Chinese Traditional Pharmaceutical Technology Co. Ltd. according to high performance liquid chromatography (HPLC). Vaccaria tablets (30 g; batch no. 140220), white mustard seed tablets (30 g; batch no. LY1505016), abutilon seed tablets (30 g; batch no. 160517HY), and plantago seed tablets (60 g; batch no. 151225) were obtained from Shanghai Union Dispensary Co. After determination, it was initially determined that SZF contains various major components such as geniposidic acid, sinapine thiocyanate, adenosine, vaccarin, verbascoside, and mustard acid. Among them, the content of geniposide is relatively highest, and its relative content reaches 33.28%. The structure of the active ingredients of SZF is shown in Figure 1.

### 2.4. Modeling and Administering

40 rats were fed freely for one week without any intervention, and they were randomly divided into normal group (n=10), model group (n=10), SZF group (n=10), and benzbromarone group (n=10). From the first day of the experiment, the two treatment groups and model group were intragastrically given 15 g/kg/d yeast powder and immediately intraperitoneally injected with 600 mg/kg/d dose of potassium oxonate. The modeling process lasted two weeks. The normal group was gavage with drinking water and injected intraperitoneally with saline. The treatment groups were given medicine from the end of making models. The dosage of treating rats was calculated according to the pharmacological methods of laboratory animals; therefore, the rats were fed with 20 times the adult dose/kg weight, and the rats in normal group and model group received the same amount of saline. During 2 weeks of continuous administration, the rats in model group and the treatment groups continued to be treated with yeast powder and potassium oxonate because of the tendency towards decreasing the level of serum uric acid automatically after the discontinuation of the modeling drugs.

### 2.5. Specimen Collection Method

At the end of the modeling, 0.5 ml blood was taken from the caudal vein of the rats, and serum uric acid was detected. After fourteen days of treatment, 24-h urine was collected in the metabolic cage, and the fresh urine samples were centrifuged at 3000 rpm for 15 min to remove the sediment. The rats were fasted and free to drink. Next day, the rats were anesthetized with 25% urethane to reach a certain depth, which is fixed on the operating table and paunched, then blood was drawn from abdominal aorta about 6-8 ml. The blood was standing for 2 hours before centrifugation 3000 rpm for 10 min, and the serum was separated and to be measured. After the abdominal aorta blood was drawn, the bilateral renal tissues of rats were taken. The left kidney was fixed with 90% ethanol for pathological section. The right kidney was placed in liquid nitrogen for Western blotting and real-time PCR detection.

### 2.6. Biochemical and Radioimmunoassay Test

Serum uric acid (SUA), serum creatinine (Scr), blood urea nitrogen (BUN), and urinary uric acid (UUA) were determined by automatic biochemical analyzer at clinical laboratory in Shuguang Hospital. Urinary *β*_2_-MG was determined by automatic double probe radioimmunoassay gamma ray counter at clinical laboratory in Shuguang Hospital. The activity of serum xanthine oxidase was determined according to instruction.

### 2.7. Pathological Observation of Renal Tissue

The left kidney cortex tissue of rats was fixed in 90% ethanol solution. After 24 hours, the kidney tissues were placed in an automatic dehydrating machine and were dehydrated by gradient alcohol and impregnated with wax. The tissues were embedded into paraffin blocks, a rotary microtome cut tissues paraffin into 5 um thickness of the sheet, the sheets were adhered in APES slides. The tissue sections were dyed with xylene dewaxing and gradient alcohol hydration and then stained with hematoxylin-eosin (HE). The preparation and observation of the 1×1×1 mm^3^ renal tissue specimens were completed in the electron microscope laboratory of Shanghai University of Traditional Chinese Medicine.

### 2.8. Immunohistochemistry

The renal tissue slices were deparaffinised and hydrated and then treated in microwave for antigen retrieval in citric acid buffer. 3% hydrogen peroxide inactivated the endogenous enzyme, and 5% bovine serum albumin (BSA) has been used to block antigen. Primary antibodies have been added dropwise to the tissue slices and incubated at 4°C overnight. HRP-conjugated secondary antibodies were incubated for 30 min at room temperature. Sections were observed through a microscope (Eclipse 80i, Nikon, Japan) after DAB color reaction. The primary antibodies used for immunohistochemistry were anti-rOAT1 (dilution 1:50), anti-rOAT3 (dilution 1:50).

### 2.9. Western Blot

Approximately 20 mg of frozen rat kidney tissues was homogenised in 200 *µ*l of RIPA buffer and then centrifuged at 12,000 rpm for 10 min. The protein concentrations of the supernatants were measured according to the BCA method. Total proteins were denaturalized in boiling water for 5 min. Equal amounts of total protein were separated onto 10% SDS-PAGE and electrophoretically transferred to a polyvinylidenedifluoride (PVDF) membrane (Millipore, Shanghai, China) which preactivated with methanol in the transferring buffer. Membranes were blocked with 5% skim milk for 2 h and incubated overnight with specific primary antibodies at 4°C. Immunoreactive bands were detected using HRP-conjugated goat anti-rabbit IgG as the secondary antibody (1:5000) (Jackson, Shanghai, China). Immunoreactive bands were visualised using a phototope-horseradish peroxidase Western blotting detection system (Cell Signaling Technologies, Beverly, MA) and quantified through densitometry with Molecular Analyst (Bio-Rad Laboratories, Hercules, CA). Primary antibodies included rabbit polyclonal antibodies against rOAT1 (dilution 1:1000) and rOAT3 (dilution 1:500).

### 2.10. Real-Time PCR

Total RNA was isolated from individual rat kidneys with Triol method. Reverse-transcribed cDNA was obtained using commercial kits. The primers sequences are as follows: rOAT1 forward: CATTGCAATCAACTGCATGACACTA; reverse: AGGAACTGGCCCAGGCTGTA; rOAT3 forward: GCTGGATCTACAACAGCACCAGAG; reverse: TGCCTGCCATGAAGATCGAC; GAPDH forward: TGCACCACCAACTGCTTAG; reverse: GATGCAGGGATGATGTTC. All primer sequences were checked in GenBank to avoid inadvertent sequence homologies. They were designed and synthesised by Sangon Biotechnology (Shanghai, China). Reactions were performed using SYBR Green PCR master mix (Applied Biosystems) in a BioRadiCycleriQ Detection System. As an internal control, GAPDH levels were quantified in parallel with the target genes. Gene expression was calculated using the 2-DeltaDelta C(T) method.

### 2.11. Statistical Analysis

The data is expressed as mean ± standard deviation (SD). One-way analysis of variance (ANOVA) was used for statistical analysis. Comparison between two groups was made through Student-Newman-Kuel's test and* p*<0.05 was considered statistically significant. Data were analyzed by statistical software SPSS18.0.

## 3. Results

### 3.1. SZF Reduces Effectively Serum Uric Acid Level in Hyperuricemic Rats

At the end of experiment, the rats in each group which suffered from modeling died in a different number, and the left rats were shown in Table 1. The serum uric acid levels of model group were significantly higher than those of each group (*p*<0.01). The results of serum uric acid in each group before and after treatment were shown in Table 1. After treatment, the serum uric acid levels of the model group were significantly higher than two medication groups (*p*<0.01) and the normal group (*p*<0.01). There was no statistically significant difference between benzbromarone group and SZF group on uric acid lowering effect (*p*>0.05).

At the second weekend of the treatment, the results of renal function in each group were shown in Table 2. Compared with the normal group, the level of serum creatinine in the model group was not significantly increased (*p*>0.05). Compared with the model group, there was no significant difference in serum creatinine between SZF group and benzbromarone group (*p*>0.05). And there was no difference in serum creatinine between the two treatment groups (*p*>0.05). Compared with the normal group, there was no significant difference in blood urea nitrogen level in model groups (*p*>0.05) and compared with the model group, no statistical significance in groups exists (*p*>0.05). The effect of SZF group was similar to benzbromarone group (*p*>0.05). This study suggests that yeast powder by intragastric gavage combined with potassium oxonate by intraperitoneal injection elevates serum uric acid level in rats, and the therapeutic efficacy of TCM formula of SZF and benzbromarone is clear and remarkable. The modeling methods influence renal function scarcely, so it is suitable for the establishment of hyperuricemic rat model that has no uric acid nephropathy.

### 3.2. SZF Increases Uric Acid Excretion and Reduces Uric Acid Production in Hyperuricemic Rats

The concentration of urinary uric acid and total uric acid excretion volume in 24 hours of laboratorial rats were shown in Table 3. Compared with the normal group, 24-h urine volume in each group increased significantly (*p*<0.01), while the urine volume of the treatment groups was more than that of the model group (*p*<0.01), but there was no difference between the SZF and benzbromarone group (*p*>0.05). The urinary uric acid excretion of model group and the treatment groups increased compared with the normal group (*p*<0.01), the excretion of the treatment groups was more than that of the model group (*p*<0.01), and there was no difference between SZF and benzbromarone group (*p*>0.05). As far as the total excretion volume in 24 hours, the same trend emerged as concentration of urinary uric acid, and SZF and benzbromarone could significantly augment the excretion of urinary uric acid (*p*<0.01). This part of the study shows that the excretion of uric acid is enhanced from kidneys under the condition of hyperuricemia, but the level of serum uric acid does not reduce in spite of the increase of urinary uric acid excretion without drug intervention. SZF and benzbromarone both have good effect on lowering uric acid, and the performance of both had no difference, suggesting SZF can promote the excretion of uric acid to reduce serum uric acid.

The activity of serum XOD in each group was shown in Table 4. Compared with the normal group, the activity of serum XOD in the model group was significantly increased (*p*<0.01). Compared with the model group, the serum XOD activity of rats in treatment group decreased significantly (*p*<0.01), and the groups between SZF and benzbromarone have no statistical difference (*p*>0.05). This part of the study indicated that SZF can not only promote the excretion of uric acid, but also lessen serum uric acid by inhibiting the formation of uric acid, so its targets on reducing uric acid levels were more diversified compared with benzbromarone.

### 3.3. SZF Protects Renal Tubules from Injury in Hyperuricemic Rats

The urinary *β*_2_-MG is a relatively recognized marker of tubular reabsorption function in recent years, and the excretion of *β*_2_-MG in each group was shown in Table 5. Compared with the normal group, the excretion of urinary *β*_2_-MG in the model group increased to about 2 times of that in the normal group (*p*<0.05). Compared with the model group, the level of urinary *β*_2_-MG in the treatment groups was decreased significantly (*p*<0.05) and there was no difference in the two treatment groups (*p*>0.05). The study suggests that although there is no damage on renal function of hyperuricemia, there still may be an injury of renal tubule, and to a certain extent, SZF and benzbromarone play a role of protection of renal tubular function.

**Figure 2:**
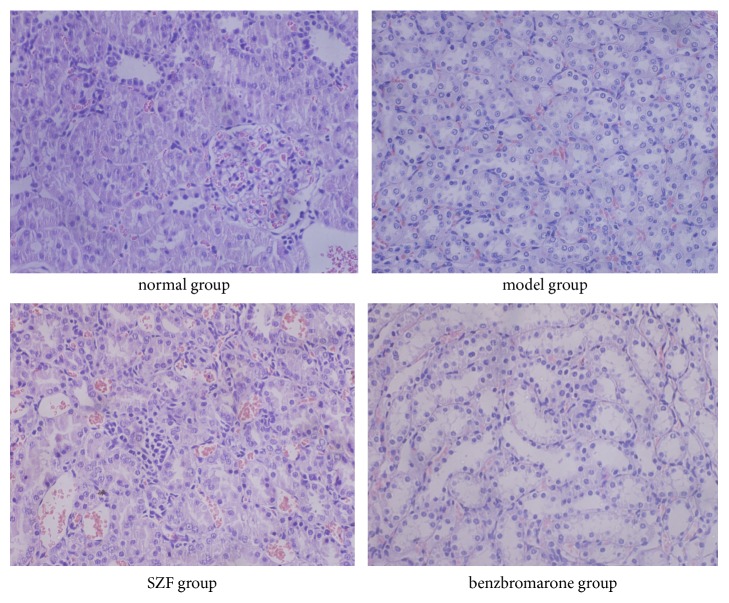
SZF improved renal pathological morphology in the hyperuricemic rats. H&E staining results of the renal tissue (magnification 100×).

The pathological staining also confirmed the above results that renal tubular injury was observed in the model group of hyperuricemic rats. Under the light microscope, the construction of renal tissue showed normal shape in normal group. However, local renal proximal tubular epithelial cell swelling, vacuolar degeneration, interstitial hyperemia, inflammatory cell infiltration occasionally, and no obvious abnormalities in glomeruli of model group were found. The groups of SZF and benzbromarone had no significant change in renal glomerular and interstitial infiltration of inflammatory cells loss, kidney swelling, and vacuolar degeneration of proximal tubular epithelial cells reduced (Figure 2). This part of the study indicated that SZF can improve renal tubular-interstitial pathological injury in hyperuricemic rats.

### 3.4. SZF Upregulated the Protein and Gene Expression of rOAT1 and rOAT3 in Hyperuricemic Rats

**Figure 3:**
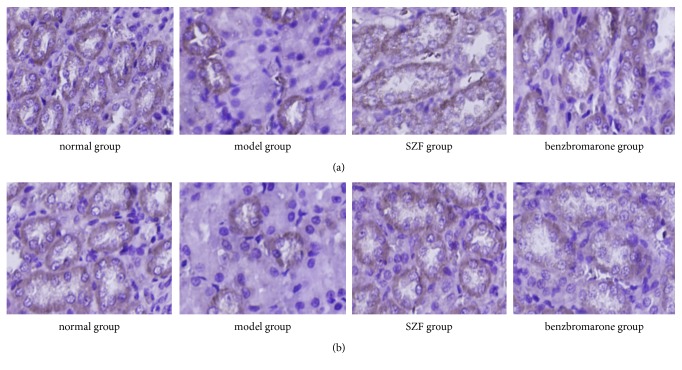
SZF upregulated the protein expression of rOAT1 and rOAT3 in hyperuricemic rats. (a) rOAT1 immunohistochemical staining (magnification 200×); (b) rOAT3 immunohistochemical staining (magnification 200×).

**Figure 4:**
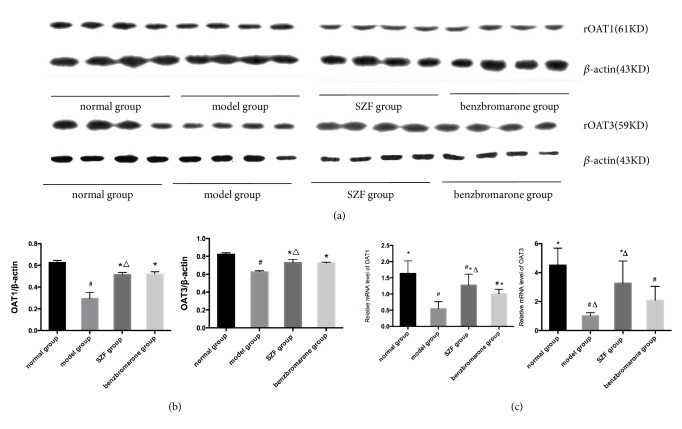
SZF upregulated the protein and gene expression of rOAT1 and rOAT3 in hyperuricemic rats. Protein levels were determined by Western blotting, were quantified through densitometry, and are expressed as the optical density ratio to *β*-actin. mRNA levels were determined through real-time PCR. Data are expressed as the mean ± SD (n = 10,7,8,7). ^#^*p*<0.05 versus the normal group; ^*∗*^*p*<0.05 versus the model group; ^△^*p*>0.05 versus the benzbromarone group.

The immunohistochemical staining of rOAT1 and rOAT3 in rats' kidneys showed that rOAT1 and rOAT3 were expressed in proximal tubular epithelial cells in each group (Figure 3); immunohistochemistry and Western blot (Figures 4(a) and 4(b)) semiquantitative results showed that the expressions of rOAT1 and rOAT3 in the model group were both significantly lower than normal group (*p*<0.01). Compared with the model group, the protein expressions of rOAT1 and rOAT3 were significantly increased in SZF group and benzbromarone group (*p*<0.01) and there was no difference between SZF group and benzbromarone group (*p*>0.05). Quantitative analysis of relative mRNA detection of rOAT1 and rOAT3 by real-time PCR (Figure 4(c)) showed that the level of gene transcription decreased significantly in model group (*p*<0.01), SZF and benzbromarone can upregulate tremendously the mRNA expression of rOAT1 and rOAT3 (*p*<0.01), and there was no difference in the two treatment groups (*p*>0.05). The study suggests that the main mechanism of uric acid excretion disorder in hyperuricemic rats maybe reduces anion transporter expression in renal tubular epithelial cell; meanwhile, SZF and benzbromarone can promote the excretion of uric acid by upregulating the gene and protein expression of rOAT1 and rOAT3.

## 4. Discussion

Our previous repeated experiments suggest that hyperuricemic rats can be induced by the dose of 15 g/kg/d and the concentration of 2 g/ml/d yeast powder intragastric gavage and the potassium oxonate 600 mg/kg/d once a day by intraperitoneal injection for two weeks. Compared with the normal group, the serum uric acid of rats in the model group is significantly increased (233.2±23.9 versus 64.7±5.4) (*p*<0.01). After the discontinuation of the modeling drug, the serum uric acid tended to decrease, so the model group and the treatment groups have to be given the yeast powder and potassium oxonate continuously to keep the model stability during the period of administration. After the medication, the serum uric acid of the model group was still much higher than that of the normal group (*p*<0.01). Therefore, the hyperuricemic model was successfully established which was evaluated with the serum uric acid value as the main index. The pathological results showed that no uric acid crystals or calculus were found in rats' kidneys, no renal tubular blockage and necrosis, no glomerular obvious abnormalities in all groups suggesting that the model induced by yeast powder and potassium oxonate caused slight renal pathological damage and no obvious abnormal renal function in rats. Therefore, this method is similar to the human high protein diet, which leads to hyperuricemia induced by purine nucleotide metabolic disorder. So, it is closer to the mode of human primary hyperuricemia. However, for this model, the duration of serum uric acid and the time point of pathological damage to renal tissue are still worth further investigation.

ShiZhiFang as an invention patent of the State Intellectual Property Office (patent authorization No. ZL200510026468.9) is the extractive harvest of two generation experts clinical experience in the Department of Nephrology in Shuguang Hospital Affiliated to Shanghai University of Traditional Chinese Medicine, showing its curative effect of clinical application, provided the solid evidence for this research. Considering the reason for the formation of hyperuricemia clinically is uric acid excretion disorder, so we adopt recognized uricosuric agent benzbromarone as a positive control drug, and its mechanism is the inhibition of renal tubular reabsorption of uric acid.

However, it has not been widely used in clinic because of the risk of urinary calculi formation and the long-standing knowledge of liver and kidney damage [19]. After SZF intervention, the serum uric acid level significantly decreased; contrarily, 24-h urine volume, urinary uric acid concentration, and uric acid excretion in 24-h urine were significantly increased, suggesting SZF not only effectively reduces serum uric acid but also promotes uric acid excretion in hyperuricemic model rats. But the experimental data manifest that after modeling and administration of hyperuricemic rat model, the renal function had no obvious abnormalities, suggesting that the model belongs to the primary pathological stage of hyperuricemia early, so this model has not yet confirmed whether SZF has a protective effect on renal function in rats, and it still needs to be clarified by further study.

The reabsorption function of renal tubules can be assessed by quantitative determination of small molecular proteins in urine. *β*_2_-MG is a relatively recognized marker of tubular reabsorption in recent years. S Liabeuf [20] and other researchers have confirmed that there is a strong correlation between the level of *β*_2_-MG and glomerular filtration rate in different stages of CKD. Hettinga YM et al. [21] demonstrated that urinary *β*_2_-MG and serum creatinine levels are sensitive and simple methods for diagnosing tubulointerstitial nephritis. In this study, the rats suffered modeling and treatment, serum uric acid and urinary *β*_2_-MG were elevated, and the pathological results showed occasionally local inflammatory cells and renal tubular epithelial cell swelling infiltration. In a cross-sectional study [22] which experienced a period of one year, Kazuko Suzuki et al. detected that urinary albumin and *β*_2_-MG as markers of glomerular and tubular injury and serum uric acid level are an independent risk factor of proteinuria in the crowd without renal injury, confirming that uric acid leads to glomerular injury in the general population, and there was no gender difference. In consideration of the excretion of urinary *β*_2_-MG being related to tubulointerstitial injury, therefore, whether or not hyperuricemia affects tubulointerstitial related to glomerular injury needs to be further investigated.

We found that the activity of serum XOD in model group was significantly increased after modeling. Potassium oxonate is a uricase inhibitor, as chemical inducers can inhibit enzyme activity and inhibit uric acid decomposition, thereby increasing the level of serum uric acid in the body [23, 24]. When a large dose of yeast is into the body, it can interfere with the normal metabolism of purine, and purine metabolic disorder accelerates the production of uric acid [25]. While the excess uric acid cannot be broken down by the presence of uricase inhibitors. This process may lead to an increase in xanthine oxidase activity, resulting in large amounts of uric acid. SZF may reduce the formation of uric acid through inhibiting the biosynthesis of XOD. Benzbromarone group compared with the model group also had significant difference, but there is no direct evidence to prove its inhibitory effect on XOD activity, and we presumed that benzbromarone reduces serum uric acid concentration, through which the regulation of XOD network was indirectly affected. Maryam, Abooali et al. [26] suggest that XOD can be activated by proinflammatory factors, and a large number of studies have shown that uric acid can activate a variety of inflammatory signals such as NF-kappa B [27] and NLRP3[28], leading to the release of proinflammatory factors. Thereby whether benzbromarone improves the inflammatory state by lowering the uric acid level and suppresses XOD activity remains to be confirmed by further studies.

The kidney is the main excretory organ of uric acid. Uric acid can be freely filtered in the glomerulus, so the reabsorption and secretion of uric acid through the renal tubule play an important role in regulating the level of serum uric acid. The process is accomplished by different transporters. Current studies have shown that OAT1 and OAT3 play a key role in uric acid secretion. OAT1 was expressed in the basolateral membrane of renal proximal tubule cells, and OAT3 was expressed in the proximal tubule and also expressed in the distal tubules [29, 30].

The mechanism of OATs transport uric acid is divided into two groups: basal lateral and free lateral of renal proximal tubule epithelial cell. The basal side OATs bind to the organic anion and actively transport into the cell by reverse concentration gradients and electrical gradients; the energy supply originates from the Na+ concentration gradient and depends on two-carboxylic acid antrorse suppression and reverse stimulation process [31]. Eraly et al. [32] found that the ability of urate secretion in renal tubules of OAT1 knockout mice was markedly diminished. Bakhiya et al. [33] found that OAT3 excreted uric acid through the exchange of organic ions with two-carboxylic acids, similar to the function of OAT1, both suggesting that OAT1 and OAT3 are involved in the secretion of uric acid. This study shows that the molecular mechanism of SZF in treating hyperuricemia is closely related to upregulation of the protein and gene expression of rOAT1 and rOAT3. In addition to the transportation of uric acid, they also play an important role in the excretion of metabolites and poisonous substance [34]. A number of studies have demonstrated that the organic anion transporters OAT1 and OAT3 are targeted drug transporters [35–37]. At the same time, it suggests that the drugs that enhance the expression of rOAT1 and rOAT3 as targets can manifold the excretion of urate and lessen the level of serum uric acid, which provides a theoretical basis for the development of new drugs for the treatment of hyperuricemia. This study has proved that SZF regulates OAT; based on this, we consider what compounds in SZF might be the substrate for OAT. A research has showed both hydrophobic and charged interactions contribute to OAT1 binding, and bigger di-anions manifest greater potency for OAT1 [38]. Another study shows that in addition to molecular charge, organic anion transporters were found to prefer interacting with planar structures. Moreover, compared with OAT1 ligands, OAT3 ligands possess more acyclic tetravalent bonds and have a more zwitterionic/cationic character [39]. Because there is no literature supporting the role of SZF and its major components as a substrate for OAT binding, through these two reports, we speculate that one or more components of SZF are hydrophobic di-anions and their molecular zwitterionic/cationic character.

Numerous studies reported the effect of traditional Chinese medicine on uric acid transport in hyperuricemia model, such as nuciferine [40], Smilacis Glabrae Rhizoma [41] and TCM compound of Siwu decoction [42], and Er-miao-san [17]. Tianqiao Yong et al. [43] found that the ethanol and water extracts of Ganoderma applanatum showed remarkable hypouricemia activities, rendering a substantial decline in serum uric acid level and the enhanced urine uric acid levels. Unlike SZF, almost no suppressing effect was observed on the XOD activities. Compared to the hyperuricemia control, OAT1 was elevated remarkably in mice drugged with G. applanatum, while GLUT9 was significantly decreased. Similar to benzbromarone, the ethanol extracts decreased the URAT1 protein levels significantly, while the water extracts did not display a similar effect. Thus, G. applanatum produced outstanding hypouricemic effects, mediated by renal OAT1, GLUT9, and URAT1 and gastrointestinal CNT2 that might elevate urine uric secretions and decline in the absorption of purine in the gastrointestinal tracts. In contrast to the above report, Yong T et al. [44] found the ethanol and water extracts of Armillaria mellea exhibited excellent hyperuricemic control because of hypouricemic actions. Moreover, they detected that A. mellea possess some inhibitory effect on XOD activity. Compared with hyperuricemic control, protein expressions of OAT1 were significantly elevated in A. mellea-treated mice. The levels of GLUT9 expression were significantly decreased by water extracts. Both the ethanol and water extracts downregulated CNT2 proteins in the gastrointestinal tract in hyperuricemic mice. Further research concluded that hydrogen bond, Pi-Pi, and Pi-sigma interactions might play important roles for their orientations and locations in XOD inhibition. The above studies have described that traditional Chinese medicine can reduce uric acid levels in the body by inhibiting the inflammatory state of hyperuricemia or regulating the expression of uric acid transporter or regulating XOD activity, or through a combination. The anti-inflammation effect of SZF has been reported in our previous study [45]. This article explores its effect on uric acid metabolism ulteriorly. We estimated that SZF has an effect on XOD activity and renal anion transporter expression; however, we did not test its effect on intestinal uric acid transporters (CNT2) and GLU and organic cation transporters (OCTs). It cannot be concluded that SZF affects intestinal filtration of uric acid and GLU and OCTs expression.

In addition, we have not detected the way that main active ingredients in SZF interact with these proteins. But there are published work supporting that OAT1 and OAT3, on the basolateral membrane of epithelial cells, transport uric acid from the renal interstitial into tubular epithelial cells. The transfer power might come from the oxygen glutarate concentration gradient intracellularly and interstitially [46]. The expression of OAT1 and OAT3 genes is coordinately regulated. In mice, deletion of either one of the pairs results in reduced renal expression of the other transporter. In the proximal tubule of the kidney, which are apically localized mediating the absorption from glomerular ultrafiltrate, the OAT1/OAT3 paired genes are basolaterally localized and mediate the initial uptake of organic anions (including urate) from the blood [47]. Therefore, we speculated that the process of the increased expression of OAT1 and OAT3 in kidneys of rats with hyperuricemia is that SZF may regulate the paired genes of OAT1/OAT3 to make their expression in the kidneys tend to increase similarly. Moreover, we hypothesize that the metabolites of SZF in the body may affect the oxoglutarate concentration gradient to increase OAT1 and OAT3 to transport uric acid into renal tubular epithelial cells. The decrease of uric acid concentration in renal interstitium also reduces the destructive effect of uric acid on renal tubular epithelial cells and to a certain extent protects the structure and function of OAT1/OAT3. Of course, these are worth exploring in future studies.

This study also testifies that there is pathological damage of renal tissue in the early stage of hyperuricemia. With the persistent development of the lesion, the pathological stage may progress into uric acid nephropathy, suggesting that simple hyperuricemia should be early intervened to reduce pathological damage. The pathogenesis of hyperuricemia is complicated, and a variety of urate transporters are involved in the reabsorption and secretion of uric acid in epithelial cells of the proximal tubule [48]. The study of pathogenesis and treatment about hyperuricemia should be combined with multifarious related factors for further exploration.

## Figures and Tables

**Table 1:** SUA levels in each group (*μ*mol/L, mean ± SD).

Groups	N	Treatment	SUA
normal group	10	0th week	64.7±5.4
		2nd weekend	71.9±13.6
model group	7	0th week	233.2±23.9^☆^
		2nd weekend	267.2±26.3^#^
SZF group	8	0th week	198.9±20.2^☆^
		2nd weekend	148.6±10.4^*∗*△^
benzbromarone group	7	0th week	208.3±14.0^☆^
		2nd weekend	153.1±10.8^*∗*^

^☆^ *p*<0.01 versus the normal group (before treatment); ^#^*p*<0.01 versus the normal group (after treatment); ^*∗*^*p*<0.01 versus the model group (after treatment); ^△^*p*>0.05 versus the benzbromarone group (after treatment).

**Table 2:** Renal function of rats in each group (mean ± SD).

Groups	N	Scr (*μ*mol/L)	BUN(mmol/L)
normal group	10	54.9±10.54	5.97±1.36
model group	7	55.4±7.34^#^	7.00±1.38^#^
SZF group	8	60.62±12.07^*∗*△^	6.85±0.71^*∗*△^
benzbromarone group	7	62.71±15.95^*∗*^	6.62±0.88^*∗*^

^#^ *p*>0.05 versus the normal group; ^*∗*^*p*>0.05 versus the model group; ^△^*p*>0.05 versus the benzbromarone group.

**Table 3:** UUA excretion of rats in each group (mean ± SD).

Groups	N	24-h urine volume(ml)	UUA (*μ*mol/L)	Total UUA excretion (*μ*mol/24h)
normal group	10	10.0±0.78	204.06±1.74	2.05±0.23
model group	7	13.0±0.65^#^	331.96±9.94^#^	4.31±0.22^#^
SZF group	8	16.4±0.56^*∗*△^	427.44±23.7^*∗*△^	7.03±0.44^*∗*△^
benzbromarone group	7	17.1±0.67^*∗*^	426.27±5.47^*∗*^	7.28±0.25^*∗*^

^#^ *p*<0.01 versus the normal group; ^*∗*^*p*<0.01 versus the model group; ^△^*p*>0.05 versus the benzbromarone group.

**Table 4:** Serum XOD activity of rats in each group (mean ± SD).

Groups	N	XOD (U/L)
normal group	10	10.27±1.16
model group	7	13.60±0.53^#^
SZF group	8	11.56±0.56^*∗*△^
benzbromarone group	7	12.24±0.50^*∗*^

^#^ *p*<0.01 versus the normal group; ^*∗*^*p*<0.01 versus the model group; ^△^*p*>0.05 versus the benzbromarone group.

**Table 5:** Urinary *β*_2_-MG of rats in each group (mean ± SD).

Groups	N	*β*_2_-MG (*μ*g/ml)
normal group	10	38.66±27.03
model group	7	66.59±36.78^#^
SZF group	8	49.37±32.92^*∗*△^
benzbromarone group	7	36.73±32.58^*∗*^

^#^ *p*<0.05 versus the normal group; ^*∗*^*p*<0.05 versus the model group; ^△^*p*>0.05 versus the benzbromarone group.

## Data Availability

The data used to support the findings of this study are available from the corresponding author upon request.
